# Supplementary figures and images for: Secondary metabolites of Hülle cells mediate protection of fungal reproductive and overwintering structures against fungivorous animals
Source: eLife. 2021 Oct 12;10:e68058. doi: 10.7554/eLife.68058 (PMC8510581; doi:10.7554/eLife.68058)

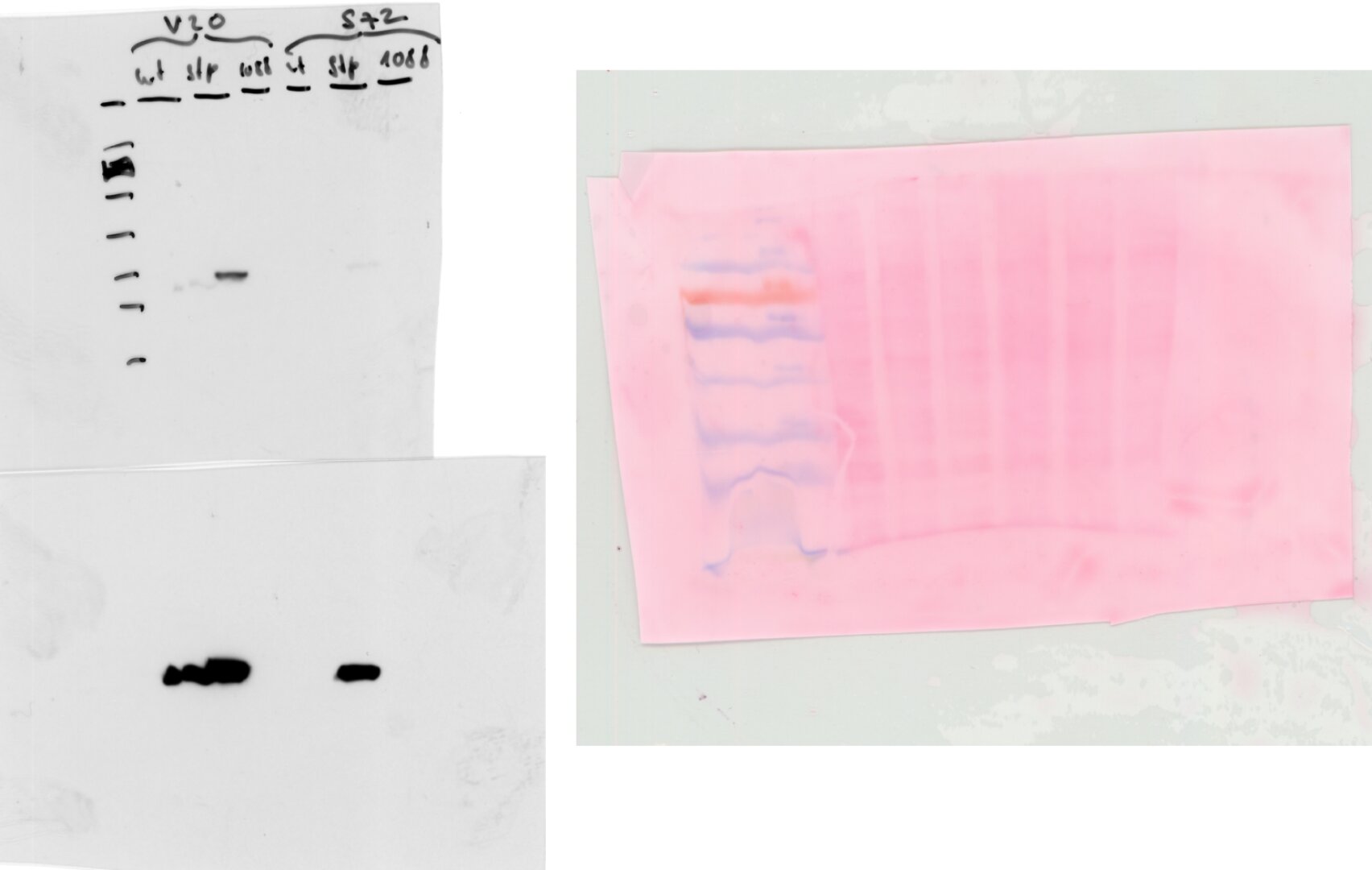

Supplement: Figure 1—source data 1. [file elife-68058-fig1-data1.jpg]

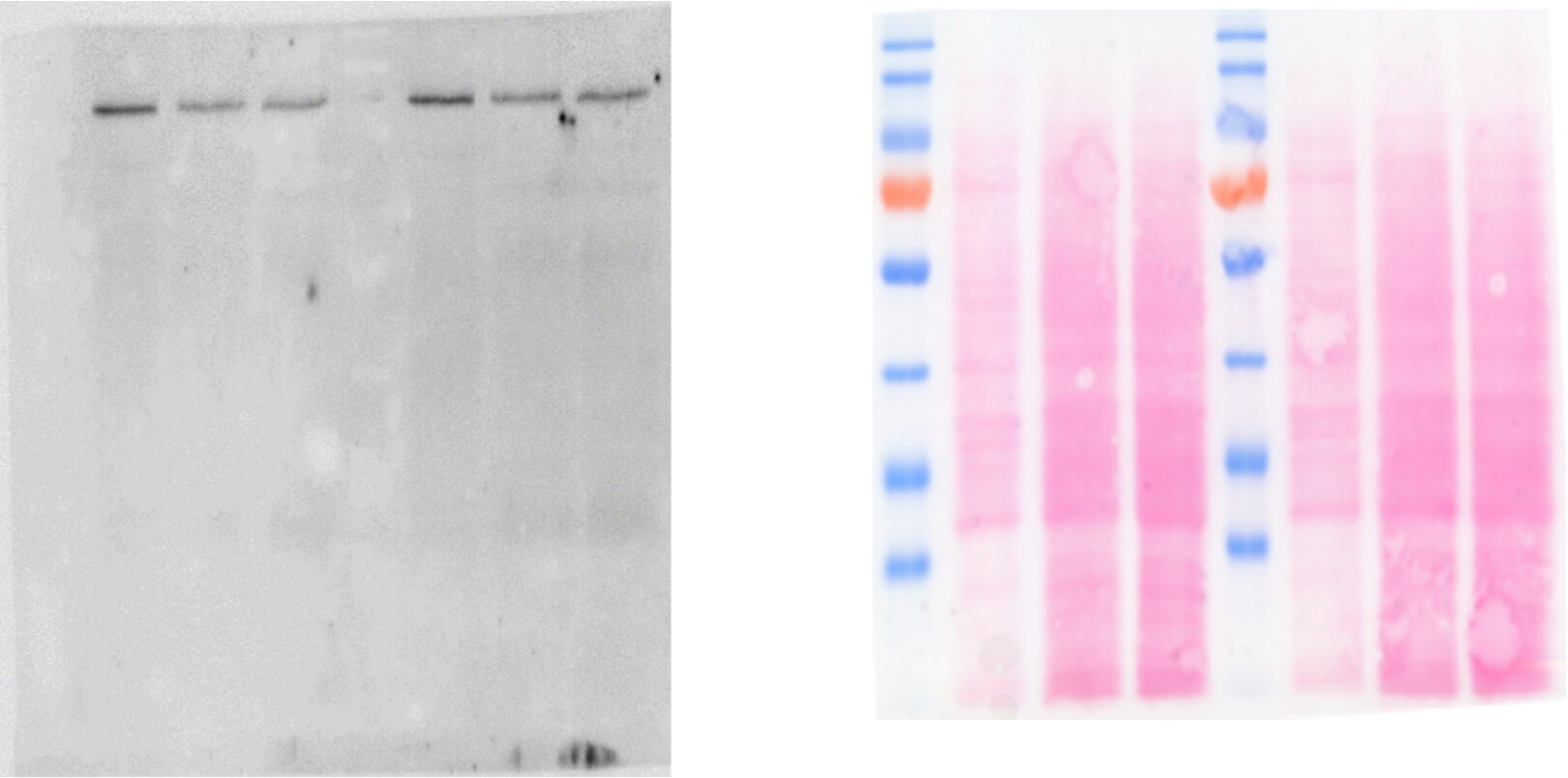

Supplement: Figure 1—source data 2. [file elife-68058-fig1-data2.jpg]

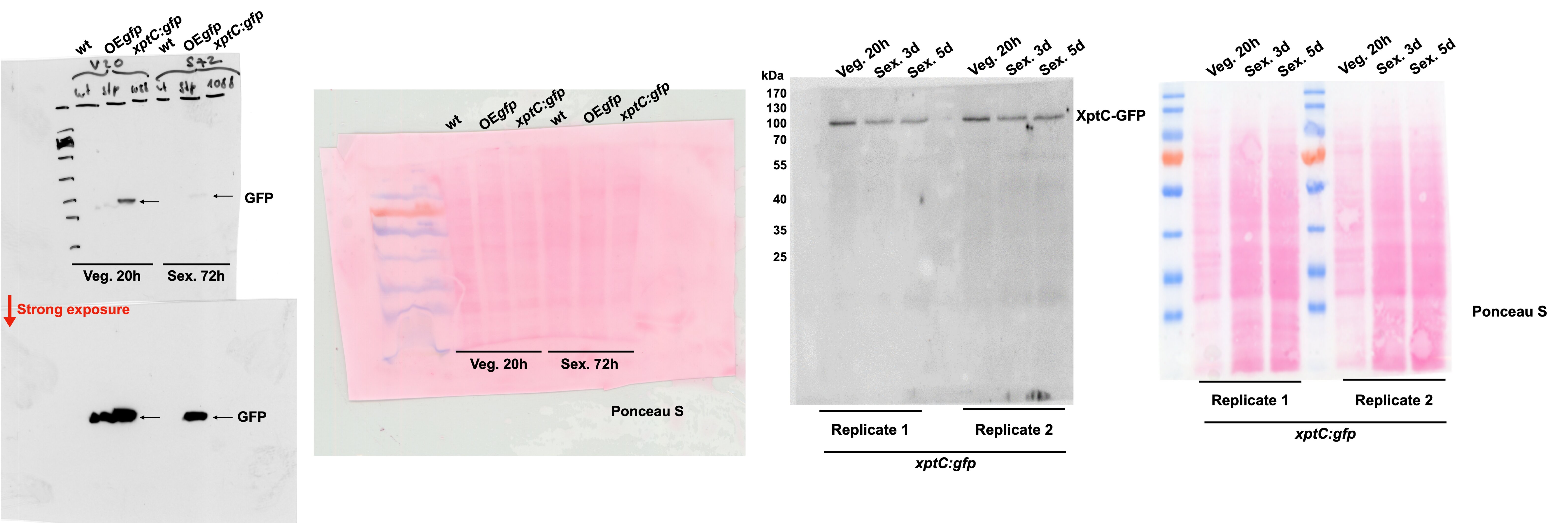

Supplement: Figure 1—source data 3. [file elife-68058-fig1-data3.jpg]
