# Supplementary material for: Secondary metabolites of Hülle cells mediate protection of fungal reproductive and overwintering structures against fungivorous animals
Source: eLife. 2021 Oct 12;10:e68058. doi: 10.7554/eLife.68058 (PMC8510581; doi:10.7554/eLife.68058)
Supplement: Supplementary file 1. [file elife-68058-supp1.docx]

**Supplementary File 1. LC-MS analysis revealed 24 proteins that were exclusively produced in both sexual mycelia and Hülle cells.**

| **Protein ID (name)** | **Description (from FungiDB)** | **3 d** | | **5 d** | | **7 d** | | |
| --- | --- | --- | --- | --- | --- | --- | --- | --- |
|  |  | **sex. mycelia** | **Hülle cells** | **sex. mycelia** | **Hülle cells** | | **sex. mycelia** | **Hülle cells** |
| AN7349 (MutA) | α-1,3-glucanase (mutanase) activity, involved in carbohydrate catabolism | 35 | 3 | 134 | 43 | | 43 | 28 |
| AN0941 (AgdE) | α-1,4-glucosidase | 3 | 6 | 173 | 32 | | 63 | 24 |
| AN3790 (AgnB) | Putative α-1,3-glucanase | 7 | - | 52 | 18 | | 14 | 17 |
| AN9443 | Has domain(s) with predicted catalytic activity and role in carbohydrate metabolic process | 11 | - | 39 | - | | 18 | 11 |
| AN7345 (AgdC) | α-glucosidase | 57 | 6 | 173 | 32 | | 63 | 24 |
| AN11143 (GlaA) | Putative glucoamylase with a predicted role in starch metabolism | 16 | - | 21 | 4 | | 13 | - |
| AN11049 | NmrA-like domain containing protein | 20 | - | 118 | 35 | | 82 | 49 |
| **AN10023 (MdpL)** | Member of the monodictyphenone (*mdp*) secondary metabolite biosynthesis gene cluster | 121 | 25 | 98 | - | | 34 | 42 |
| AN7999 | Putative oxidoreductase | 21 | - | 147 | 8 | | 16 | 47 |
| **AN7998 (XptC)** | GMC oxidoreductase | 54 | 7 | 48 | 6 | | 26 | - |
| **AN12402 (XptB)** | Prenyltransferase | 52 | 5 | 19 | - | | - | 8 |
| **AN10022 (MdpH)** | Protein with homology to the DUF 1772 superfamily | 27 | - | 2 | 5 | | 27 | 6 |
| **AN0150 (MdpG)** | Polyketide synthase | 12 | 4 | - | 3 | | - | - |
| AN7812 (StcN) | Putative versicolorin B synthase with a predicted role in sterigmatocystin/aflatoxin biosynthesis | 32 | 3 | 9 | 4 | | 10 | - |
| AN7641 | Putative copper amine oxidase | 22 | 6 | 31 | 2 | | - | - |
| AN6314 | Has domain(s) with predicted oxidoreductase activity and role in metabolic process | 13 | 5 | 150 | 27 | | 111 | 48 |
| AN8203 | Ortholog(s) have NAD+ synthase (glutamine-hydrolyzing) activity, glutaminase activity, role in NAD biosynthetic process and cytosol, nucleus localization | 11 | - | 8 | 5 | | - | - |
| AN1142 | Has domain(s) with predicted UDP-N-acetylmuramate dehydrogenase activity, flavin adenine dinucleotide binding, oxidoreductase activity and role in oxidation-reduction process | 24 | - | 11 | 4 | | - | - |
| AN11897 | Has domain(s) with predicted RNA binding, ribonuclease T2 activity | 6 | - | 19 | 6 | | 10 | - |
| AN5669 | Putative succinyl-CoA:3-ketoacid-coenzyme A transferase | 19 | 8 | 42 | - | | - | 2 |
| AN10977 | Ortholog(s) have extracellular region localization | 14 | - | 4 | 6 | | - | - |
| AN6930 | Has domain(s) with predicted catalytic activity, pyridoxal phosphate binding, transaminase activity | 2 | - | 14 | - | | - | 2 |
| AN5488 | Protein of unknown function | 21 | 2 | - | 20 | | - | - |
| AN1863 | Protein of unknown function | 8 | - | - | - | | 2 | 4 |

A. nidulans wildtype A4 was cultivated on agar plates under sexual conditions for three, five and seven days. Hülle cells were enriched from plates. Proteins were identified by LC-MS. Only proteins identified in two or more biological replicates and with two or more peptides per protein were considered for the analysis. Numbers represent the average of spectral counts from three biological replicates. Proteins in bold are encoded by mdp/xpt genes. Source data are provided as a Source data file.

Table S1-Source data 1
