# Supplementary material for: Secondary metabolites of Hülle cells mediate protection of fungal reproductive and overwintering structures against fungivorous animals
Source: eLife. 2021 Oct 12;10:e68058. doi: 10.7554/eLife.68058 (PMC8510581; doi:10.7554/eLife.68058)
Supplement: Supplementary file 2. [file elife-68058-supp2.docx]

**Supplementary File 2.** **Secondary metabolites produced by the mdp/xpt genes in A. nidulans sexual development identified by LC-MS**

| **ID** | **Metabolite** | **Retention time(min)** | **Detected as** | **Sum formula** | **Detected exact mass** | **Calculated exact mass** | **Confirmed by** | **Reference** |
| --- | --- | --- | --- | --- | --- | --- | --- | --- |
| **1** | arugosin A | 20.36 | [M-H]^-^ | C_25_H_28_O_6_ | 423.1811 | 424.1964 | A, C | (Albright et al., 2015; Pockrandt et al., 2012) |
| **2** | emericellin | 24.33 | [M-H_2_O+H]^+^ | C_25_H_28_O_5_ | 391.1896 | 408.1937 | A, B | (Sanchez et al., 2011) |
| **3** | shamixanthone | 24.52 | [M-H_2_O+H]^+^ | C_25_H_26_O_5_ | 389.1743 | 406.1780 | A, B | (Sanchez et al., 2011) |
| **4** | epishamixanthone | 25.46 | [M-H_2_O+H]^+^ | C_25_H_26_O_5_ | 389.1747 | 406.1780 | A, B | (Sanchez et al., 2011) |
| **5** | 2,ω-dihydroxyemodin | 8.96 | [M-H]^-^ | C_15_H_10_O_7_ | 301.0351 | 302.0348 | A, B | (Sanchez et al., 2011) |
| **6** | ω-hydroxyemodin | 11.48 | [M-H]^-^ | C_15_H_10_O_6_ | 285.0399 | 286.0477 | A, B | (Sanchez et al., 2011) |
| **7** | emodin | 16.09 | [M-H]^-^ | C_15_H_10_O_5_ | 269.0451 | 270.0528 | A, B, D | (Sanchez et al., 2011) |
| **8** | chrysophanol | 18.66 | [M-H]^-^ | C_15_H_10_O_4_ | 253.0503 | 254.0579 | A, B | (Sanchez et al., 2011) |
| **9** | paeciloxanthone | 20.62 | [M+H]^+^ | C_20_H_20_O_4_ | 325.1435 | 324.1362 | A, B | (Sanchez et al., 2011) |
| **10** | variecoxanthone A | 20.39 | [M-H_2_O+H]^+^ | C_20_H_20_O_5_ | 323.1270 | 340.1311 | A, B | (Sanchez et al., 2011) |

Conidia of *A. nidulans* AGB552 and *mdp*/*xpt* deletion strains were point-inoculated on MM and sexually grown for three and five days at 37°C. Extra- and intracellular metabolites were extracted and detected by LC-MS equipped with a charged aerosol detector (CAD). Only identified SMs that were detected with the CAD are given. A: Exact mass measurement; B: UV/VIS spectrum; C: MS/MS fragmentation; D: MS/MS fragmentation and retention time from commercial standard.

**References**

Albright, J. C., Henke, M. T., Soukup, A. A., McClure, R. A., Thomson, R. J., Keller, N. P., & Kelleher, N. L. (2015). Large-Scale Metabolomics Reveals a Complex Response of *Aspergillus nidulans* to Epigenetic Perturbation. *ACS Chemical Biology, 10*(6), 1535-1541. doi:<https://doi.org/10.1021/acschembio.5b00025>

Pockrandt, D., Ludwig, L., Fan, A., König, G. M., & Li, S. M. (2012). New Insights into the Biosynthesis of Prenylated Xanthones: Xptb from *Aspergillus nidulans* Catalyses an O‐Prenylation of Xanthones. *ChemBioChem, 13*(18), 2764-2771. doi: <https://doi.org/10.1002/cbic.201200545>

Sanchez, J. F., Entwistle, R., Hung, J.-H., Yaegashi, J., Jain, S., Chiang, Y.-M., Wang, C. C., & Oakley, B. R. (2011). Genome-based deletion analysis reveals the prenyl xanthone biosynthesis pathway in *Aspergillus nidulans*. *Journal of the American Chemical Society, 133*(11), 4010-4017. doi:<https://doi.org/10.1021/ja1096682>
