# Supplementary material for: Secondary metabolites of Hülle cells mediate protection of fungal reproductive and overwintering structures against fungivorous animals
Source: eLife. 2021 Oct 12;10:e68058. doi: 10.7554/eLife.68058 (PMC8510581; doi:10.7554/eLife.68058)
Supplement: Supplementary file 3. [file elife-68058-supp3.docx]

**Supplementary File 3. Fungal strains used in this study**

| Strain name | Genotype/Information | Reference |
| --- | --- | --- |
| FGSC A4 | *veA*+ | FGSC* |
| AGB552 | *pabaA1; ∆nkuA::argB; veA+* | (Bayram et al., 2012) |
| AGB596 | *^p^gpdA:sgfp:phleo^R^; pabaA1; yA2; veA+* | (Bayram et al., 2012) |
| AGB1073 | *∆laeA::six; pabaA1; ∆nkuA::argB, veA+* | This study |
| AGB1088 | *xptC:gfp; pabaA1; yA2;* Δ*nkuA::argB* | This study |
| AGB1236 | *∆mdpG::six; pabaA1; ∆nkuA::argB; veA+* | This study |
| AGB1237 | *∆mdpF::six; pabaA1; ∆nkuA::argB; veA+* | This study |
| AGB1238 | *∆mdpC::six; pabaA1; ∆nkuA::argB; veA+* | This study |
| AGB1239 | *∆mdpL::six; pabaA1; ∆nkuA::argB; veA+* | This study |
| AGB1240 | *∆mdpD::six; pabaA1; ∆nkuA::argB; veA+* | This study |
| AGB1241 | *∆xptA::six; pabaA1; ∆nkuA::argB; veA+* | This study |
| AGB1242 | *∆xptB::six; pabaA1; ∆nkuA::argB; veA+* | This study |
| AGB1243 | *∆xptC::six; pabaA1; ∆nkuA::argB; veA+* | This study |
| AGB1248 | *∆mdpG::six, mdpG:six::six; pabaA1; ∆nkuA::argB; veA+* | This study |
| AGB1249 | *∆mdpC::six, mdpC:six::six; pabaA1; ∆nkuA::argB; veA+* | This study |
| AGB1310 | *∆veA::six; pabaA1; ∆nkuA::argB, veA+* | This study |
| AGB1311 | *∆velB::six; pabaA1; ∆nkuA::argB, veA+* | This study |
| *Sordaria macrospora* Taxid5147 | wildtype isolate | (Nowrousian et al., 2010) |
| *Verticillium longisporum* VL43 | wildtype isolate | (Zeise et al., 2002) |
| *Verticillium dahliae* JR2 | wildtype isolate | (Fradin et al., 2009) |

* Fungal Genetics Stock Center (Kansas), ^R^ = resistance, ^P^ = promoter, *six* = β-recombinase recognition sequence

**References**

Bayram, Ö., Bayram, Ö. S., Ahmed, Y. L., Maruyama, J.-i., Valerius, O., Rizzoli, S. O., Ficner, R., Irniger, S., & Braus, G. H. (2012). The *Aspergillus nidulans* MAPK module AnSte11-Ste50-Ste7-Fus3 controls development and secondary metabolism. *PLoS Genetics, 8*(7), e1002816. doi:<https://doi.org/10.1371/journal.pgen.1002816>

Fradin, E. F., Zhang, Z., Ayala, J. C. J., Castroverde, C. D., Nazar, R. N., Robb, J., Liu, C.-M., & Thomma, B. P. (2009). Genetic dissection of *Verticillium* wilt resistance mediated by tomato Ve1. *Plant Physiology, 150*(1), 320-332. doi:<https://doi.org/10.1104/pp.109.136762>

Nowrousian, M., Stajich, J. E., Chu, M., Engh, I., Espagne, E., Halliday, K., Kamerewerd, J., Kempken, F., Knab, B., & Kuo, H.-C. (2010). De novo assembly of a 40 Mb eukaryotic genome from short sequence reads: *Sordaria macrospora*, a model organism for fungal morphogenesis. *PLoS Genetics, 6*(4), e1000891. doi:<https://doi.org/10.1371/journal.pgen.1000891>

Zeise, K., & von Tiedemann, A. (2002). Host specialization among vegetative compatibility groups of *Verticillium dahliae* in relation to *Verticillium longisporum*. *Journal of Phytopathology, 150*(3), 112-119. doi:<https://doi.org/10.1046/j.1439-0434.2002.00730.x>
