# Supplementary material for: Secondary metabolites of Hülle cells mediate protection of fungal reproductive and overwintering structures against fungivorous animals
Source: eLife. 2021 Oct 12;10:e68058. doi: 10.7554/eLife.68058 (PMC8510581; doi:10.7554/eLife.68058)
Supplement: Supplementary file 4. [file elife-68058-supp4.docx]

**Supplementary File 4. Plasmids employed in this study**

| Plasmid | Description | Reference |
| --- | --- | --- |
| pBluescript SK(+) | Cloning vector, *amp*^R^ | Thermo Scientific |
| pME4305 | *six:*^P^*xylP:β-rec:trpC* ^t^*:phleo*^R^*:six* | (Thieme et al., 2018) |
| pME4319 | *Swa*I*:six:*^P^*xylP:β-rec:trpC*^t^ *:phleo*^R^*:six:Pml*I | This study |
| pME4574 | *5’UTR(veA): nat*^RM^*:3’UTR(veA)* | (Thieme et al., 2018) |
| pME4605 | *5’UTR(velB): nat*^RM^*:3’UTR(velB)* | (Thieme et al., 2018) |
| pME4636 | *5’UTR(laeA):phleo^RM^:3’UTR(laeA)* | This study |
| pME4645 | 5’UTR(*xptC*)*:xptC:gfp:phleo*^RM^*:*3’UTR(*xptC*) | This study |
| pME4842 | 5’UTR(*mdpG*)*:phleo*^RM^*:*3’UTR(*mdpG*) | This study |
| pME4843 | 5’UTR(*mdpF*)*:phleo*^RM^*:*3’UTR(*mdpF*) | This study |
| pME4844 | 5’UTR(*mdpC*)*:phleo*^RM^*:*3’UTR(*mdpC*) | This study |
| pME4845 | 5’UTR(*mdpL*)*:phleo*^RM^*:*3’UTR(*mdpL*) | This study |
| pME4846 | 5’UTR(*mdpD*)*:phleo*^RM^*:*3’UTR(*mdpD*) | This study |
| pME4847 | 5’UTR(*xptA*)*:phleo*^RM^*:*3’UTR(*xptA*) | This study |
| pME4848 | 5’UTR(*xptB*)*:phleo*^RM^*:*3’UTR(*xptB*) | This study |
| pME4849 | 5’UTR(*xptC*)*:phleo*^RM^*:*3’UTR(*xptC*) | This study |
| pME4850 | 5’UTR(*mdpG*)*:mdpG:phleo*^RM^*:*3’UTR(*mdpG*) | This study |
| pME4851 | 5’UTR(*mdpC*)*:mdpC:phleo*^RM^*:*3’UTR(*mdpC*) | This study |

^P^ = promoter, ^t^ = terminator, ^R^ = resistance, *phleo*^RM^ = recyclable phleomycin resistance cassette, *six* = β-recombinase recognition sequence

**References**

Thieme, K. G., Gerke, J., Sasse, C., Valerius, O., Thieme, S., Karimi, R., Heinrich, A. K., Finkernagel, F., Smith, K., & Bode, H. B. (2018). Velvet domain protein VosA represses the zinc cluster transcription factor SclB regulatory network for *Aspergillus nidulans* asexual development, oxidative stress response and secondary metabolism. *PLoS Genetics, 14*(7), e1007511. doi:<https://doi.org/10.1371/journal.pgen.1007511>
