# Supplementary material for: Secondary metabolites of Hülle cells mediate protection of fungal reproductive and overwintering structures against fungivorous animals
Source: eLife. 2021 Oct 12;10:e68058. doi: 10.7554/eLife.68058 (PMC8510581; doi:10.7554/eLife.68058)
Supplement: Supplementary file 5. [file elife-68058-supp5.docx]

# Supplementary File 5. Primers for DNA sequence amplification and plasmid construction

| Primer | 5’-sequence-3’ |
| --- | --- |
| BD45 | ATC GAT AAG CTT GAT GTT TAA ACA AGA ATT TCC GTA TGA AGC A |
| BD46 | ACC TAT AGG CCT GAG CAG CGG CGA AAC AGG GTA CA |
| BD47 | ATA ATA TGG CCA TCT GAG CAA AAG GCG ACC ACA TCC |
| BD48 | CTG CAG GAA TTC GAT GTT TAA ACT GAC TTT GGC TGT ATA GCT |
| BD106 | GGT GGT AGC GGT GGT GTG AG |
| BD107 | CTA CTT GTA CAG TTC GTC CAT G |
| BD113 | AGG CTC AAC CTG ATA CTT ACC |
| BD114 | GCT CAC ACC ACC GCT ACC ACC GTT ACC CAG CCA GCC ATG |
| BD115 | ATT AGA TCT ATT AGA CCG CAG G |
| BD116 | CAC GTG ATG TGA TAC GGT AC |
| EFS46 | TCC CCC GGG CTG CAG GAA TTC GAT GTT TAA ACC CGA CAA GAG CAG CTT TG |
| flip1 | ACC TAT AGG CCT GAG ATT TAA ATA TCG AAT TCC TGC AGC CCG G |
| flip2 | ATA ATA TGG CCA TCT CAC GTG ATC AAG CTT ATC GAT ACC GTC G |
| JG1076 | ATA ATA TGG CCA TCT GCA GTA TGT TAA CCG GTA GTG AA |
| LL59 | ACG GTA TCG ATA AGC TTG ATG TTT AAA CCA TCG TTT TAC ACC TCC TC |
| LL60 | CAT CAC GAT TGA GCC TAT ACC G |
| LL61 | GTA TAG GCT CAA TCG TGA TGA AGC GCC TTG AAG ATG CTG AG |
| LL62 | TAT TGA CCT ATA GGC CTG AGC TAA TTA TAG TAC TCT AAT AGC CAG |
| LL139 | AGG AAT TCG ATA TTT GTT TAA ACC ATC GTT TTA CAC CTC CTC |
| LL140 | ATA GGC CTG AGA TTT TCT GAG GCG ATG GAA CCA CC |
| LL141 | ATA TGG CCA TCT CAC GCA GTA TGT TAA CCG GTA GTG A |
| LL142 | GAT AAG CTT GAT CAC GTT TAA ACC CGA CAA GAG CAG CTT TG |
| LL162 | AGG AAT TCG ATA TTT GTT TAA ACC CTT CAC TAT CAT GCG TG |
| LL163 | ATA GGC CTG AGA TTT TGT GAC TCT CAG TGC TGG TGG |
| LL164 | ATA TGG CCA TCT CAC TTA CCT TTT CTC CAA AGA TTT AGA AT |
| LL165 | GAT AAG CTT GAT CAC GTT TAA ACA TGT CCG TCA CCG TTC C |
| LL193 | AGG AAT TCG ATA TTT GTT TAA ACA CCC AAC CAC CAT CAA CC |
| LL194 | ATA GGC CTG AGA TTT TTT TGA CGC CGT ATT CGT GCT T |
| LL195 | TAT GGC CAT CTC ACT TAG ATA TAC GCA GTG CTG TAT AT |
| LL196 | GAT AAG CTT GAT CAC GTT TAA ACT CTG AAT TTT TAG ATG CGA AT |
| LL197 | AGG AAT TCG ATA TTT GTT TAA ACT CGC ACT ACC TCG GCA C |
| LL198 | ATA GGC CTG AGA TTT TGT GTC TGG TTA GAA AAT GCA CAA |
| LL199 | ATA TGG CCA TCT CAC TTA AGC GAG GCA TTG GAT GGA G |
| LL200 | GAT AAG CTT GAT CAC GTT TAA ACT ATA AAT CAA GCA TTA ACC AAG |
| LL211 | AGG AAT TCG ATA TTT GTT TAA ACA TGC AGA TCA AGC CTT ACT |
| LL212 | ATA GGC CTG AGA TTT CTT GGA GGT ACT TTT CAG ATT CTA A |
| LL213 | ATA TGG CCA TCT CAC TCT ACA CCT TGA CCA CAA CCG |
| LL214 | GAT AAG CTT GAT CAC GTT TAA ACT CTA TAT CAT CCT GGT CAA C |
| LL215 | AGG AAT TCG ATA TTT GTT TAA ACC CAT CCC CAT CCC TTT G |
| LL216 | ATA GGC CTG AGA TTT GCT GGT CAG TTT GCA TGA TGG |
| LL217 | ATA TGG CCA TCT CAC TTC CTC TCT AGA AAC TTC TCA AAT |
| LL218 | GAT AAG CTT GAT CAC GTT TAA ACA AGC AGA GGG ATA CCG C |
| LL219 | AGG AAT TCG ATA TTT GTT TAA ACA CAA GGA CCC ATT ATC GTA |
| LL220 | ATA GGC CTG AGA TTT TTT CTC ACA AGC GAA GGA TAC C |
| LL221 | ATA TGG CCA TCT CAC ATT AGA TCT ATT AGA CCG CAG GC |
| LL222 | GAT AAG CTT GAT CAC GTT TAA ACA GCT CAA ATA CTC TCG AGC |
| LL223 | AGG AAT TCG ATA TTT GTT TAA ACG GAG GCT CGC AGG CCT |
| LL224 | ATA GGC CTG AGA TTT TGG TGC TTT CCT ACC TAC CTT A |
| LL225 | ATA TGG CCA TCT CAC TTC TGC TAG ATT TAG TAG CTA AGT |
| LL226 | GAT AAG CTT GAT CAC GTT TAA ACT ACC TTT CCT CAG TAC CAA |
| LL236 | ATA GGC CTG AGA TTT CTA AGC AGC GCC TCC GTC GA |
