# Supplementary material for: Secondary metabolites of Hülle cells mediate protection of fungal reproductive and overwintering structures against fungivorous animals
Source: eLife. 2021 Oct 12;10:e68058. doi: 10.7554/eLife.68058 (PMC8510581; doi:10.7554/eLife.68058)
Supplement: Supplementary file 6. [file elife-68058-supp6.docx]

**Supplementary File 6 Primers for semi-quantification of the *mdp/xpt* cluster genes**

| Primer | 5’-sequence-3’ | Gene target | product/bp |
| --- | --- | --- | --- |
| LL307 | TGG AGA CCA GAT TGA AGA AGA A | AN0153 | 384 |
| LL308 | GGC TCA GAT ACG AAG GAG GTA A |  |  |
| LL309 | TAC ATC CTA CAC CTC CCC TCT C | *mdpA* | 305 |
| LL310 | ATC TTA TTC ACC ACC ACC AAC C |  |  |
| LL311 | ATT CGT GGC TAT GGT CTC TCA T | *mdpB* | 322 |
| LL312 | GAC TCC GTT CTC TTC CTT CTC A |  |  |
| LL313 | GTA GTA ATG CGG GGG TTG TTA G | *mdpC* | 354 |
| LL314 | GTT TTT CGC CGT TTG GAA TA |  |  |
| LL315 | TAT GGA AAG ACG GTG AAG AGC | *mdpD* | 343 |
| LL316 | GCG TGA ATC CGA AGG AGA TAA T |  |  |
| LL317 | CAC CAG ATG TAA CCA GTG TTG G | *mdpE* | 326 |
| LL318 | TTC TAA TGC TTG GCT GTC TTC A |  |  |
| LL319 | TCC TTC GTC TTT ACC CTA ATC TTG | *mdpF* | 369 |
| LL320 | CCT GCT TCT CTC GTC TCA TTT T |  |  |
| LL321 | CTT GTG TCA GTG GAG ATT GCT C | *mdpG* | 400 |
| LL322 | CTG TTT GTG AGG AGT GAA ATG C |  |  |
| LL323 | TCT CCT CTG CCT CAC TAT CCT C | *mdpH* | 361 |
| LL324 | CGT CAT CTT GCT TCT CTT CGT A |  |  |
| LL325 | TAC AGA AAA TCA GGG ACA CAC C | *mdpL* | 377 |
| LL326 | GCA GAG AGC ATA CCA CAT CAG A |  |  |
| LL327 | CAG GAC CCG ATA ACA AAA TCC | *mdpJ* | 370 |
| LL328 | TAA ACC AGC CAA TCT CAA GGA C |  |  |
| LL329 | ACG AGT TGA AGG GTA GAA ATG G | *mdpI* | 318 |
| LL330 | GGA TAA TAC TTG GGC AGG AGA C |  |  |
| LL331 | CGA AAC TGG TCC TTC TCT CCT | *mdpK* | 300 |
| LL332 | ACC TCC CAT CTG TAT CAT TTG C |  |  |
| LL333 | TCA GAT TAC CAC TCA GCC ATT G | *xptA* | 348 |
| LL334 | CAC AGT TGG GGA CAA GAC CT |  |  |
| LL335 | CTC CTG AAG CCA TTC TGT CTC A | *xptB* | 369 |
| LL336 | GTT ACG CAC GGG GAA GTA GA |  |  |
| LL337 | ACC TGG AGT TGG GTC TAA TCT G | *xptC* | 305 |
| LL338 | CAA GAA CAG TGG GAT GAA TGT C |  |  |
| LL339 | ATC AAC GGA GAA ACC ATA CCA C | AN10039 | 335 |
| LL340 | AGC GGA GGA GAG ATA AGA GAC C |  |  |
